# Longitudinal Study of IgG Antibody Levels Over 4.5 Years Until the Fifth Booster mRNA Vaccination and Breakthrough Infection: A Case Report

**DOI:** 10.7759/cureus.95359

**Published:** 2025-10-24

**Authors:** Harukazu Hirano

**Affiliations:** 1 Family Medicine Department, Koyo Seikyo Clinic, Fukui, JPN

**Keywords:** bnt162b2 mrna vaccine, covid-19 breakthrough infection, hybrid immunity, igg antibody, sars-cov-2 (severe acute respiratory syndrome coronavirus -2)

## Abstract

The differential antibody responses and persistence of immunity following booster vaccinations and breakthrough infections with Severe Acute Respiratory Syndrome Coronavirus 2 (SARS-CoV-2) remain inadequately understood. This case study examined a 71-year-old Japanese man who received his seventh dose (fifth booster) of the mRNA BNT162b2 vaccine and subsequently experienced his first breakthrough infection 80 days after vaccination. A total of twenty-three serological assays were performed to quantify anti-spike protein immunoglobulin G (IgG) antibody levels from one week to one year and eight months post-infection. Six weeks after infection, the antibody titer peaked at 76,000 AU/ml, which was 14 times higher than the baseline level (5,555 AU/ml). At one year and eight months post-infection, the titer remained at 19,000 AU/ml, representing 3.8 times the baseline and one-fourth of the peak value observed after the sixth vaccination. The decline in antibody titers after breakthrough infection was modeled using the equation f(t) = Aexp(−*t*/τ) + C. These findings suggest that humoral immune responses are maintained at elevated levels for an extended period. This case provides valuable insights into vaccination strategies for breakthrough infections.

## Introduction

Since the World Health Organization declared the novel coronavirus disease 2019 (COVID-19) pandemic in March 2020, the virus has caused multiple waves of infection worldwide. As of October 2024, infection with Severe Acute Respiratory Syndrome Coronavirus 2 (SARS-CoV-2), the etiological agent of the COVID-19 pandemic, has affected over 760 million individuals and resulted in 7.0 million fatalities worldwide [1]. However, these statistics may significantly underestimate the true infection rates owing to factors such as limited testing, asymptomatic cases, deficiencies in public health measures, and the simplification or discontinuation of infection reporting systems. The efficacy of the mRNA vaccine BNT162b2 (Pfizer-BioNTech) was initially demonstrated in randomized controlled trials [2], which subsequently led to global vaccination campaigns worldwide. Although various vaccines have been approved to mitigate the spread of COVID-19, mRNA vaccines (BNT162b2 and mRNA-1273) have been predominantly utilized in Japan.

Initially identified in South Africa in November 2021, the Omicron variant swiftly became the predominant strain and subsequently evolved into various sublineages, including BA.1 and BA.2. It is characterized by at least 37 mutations in the spike protein, which accounts for its high transmissibility and capacity to evade immune responses [3]. Current research and development efforts for next-generation vaccines against these emerging variants emphasize that these variants can diminish vaccine efficacy. These efforts have focused on the design of broad-spectrum vaccines targeting conserved regions, such as the S2 subunit, and the application of advanced development techniques, including antigen selection using artificial intelligence, structural biology, and nanoparticle technology [4].

In most cases, SARS-CoV-2 infection results in self-limiting symptoms. Nevertheless, individuals with pre-existing conditions, such as pulmonary diseases, diabetes, immunosuppression, or advanced age, are at an increased risk of developing severe and potentially fatal manifestations of the disease owing to excessive proinflammatory cytokine release [5]. Vaccination remains the most critical preventive strategy against COVID-19, even for individuals with pre-existing medical conditions, and is vital to public health. Five years into the COVID-19 pandemic, the SARS-CoV-2 virus has entered the omicron era, necessitating research on the dynamics of humoral and cellular immunity following booster vaccinations and breakthrough infections. Following COVID-19 vaccination, antibody levels increase significantly and then decline over several months. Binding antibodies, such as immunoglobulin G (IgG) targeting spike proteins or the receptor-binding domain (RBD), tend to persist for an extended duration. In contrast, neutralizing antibodies that inhibit viral infection may diminish more rapidly. While binding antibodies reflect the ability to bind to viral antigens, neutralizing antibodies function by obstructing the virus from entering cells and are strongly correlated with protective efficacy. The rate and pattern of antibody decay are influenced by factors such as vaccine type, age, and individual characteristics. In older adults who have received the BNT162b2 vaccine, there is a significant reduction in spike protein-specific IgG titers, and spike protein-specific cluster differentiation (CD)4 and CD8 T-cell immunity is limited, leading to diminished immunogenicity and vaccine efficacy [6].

Among the structural proteins of SARS-CoV-2, the spike and nucleocapsid proteins serve as the primary immunogens. The assessment of antibodies against spike receptor-binding domain (RBD) IgG is crucial for evaluating protective effects against SARS-CoV-2 infection, as it correlates with their neutralizing activity and can be readily measured [7]. Antibodies elicited by mRNA vaccines specifically target the RBD of the S1 subunit of the SARS-CoV-2 spike protein [8]. In the immune response induced by mRNA vaccines, the quantity, function, and longevity of immune memory cells are crucial, and IgG antibody levels against the SARS-CoV-2 spike protein have been examined to evaluate the humoral immune response after vaccination. Despite advancements in vaccination strategies, mathematical models have yet to be developed to analyze long-term trends in breakthrough infections following the administration of multiple booster doses.

An older adult aged 71 years experienced a breakthrough infection after receiving the seventh dose of the BNT162b2 vaccine. This case report presents a systematic investigation of IgG antibody levels over an extended period following a breakthrough infection and conducts a detailed longitudinal analysis of their dynamics. To the best of our knowledge, this case study is unique, and no similar reports exist in the literature.

## Case presentation

In this case study, the participant was a 71-year-old Japanese male healthcare worker with no underlying health conditions other than hypertension and dyslipidemia. He had no history of smoking, and his body mass index (BMI) was 21. The participant received the initial dose of the BNT162b2 vaccine in April 2021, followed by a second dose in May 2021, 21 days after the first. The third dose was administered in December 2021, 216 days after the second dose; the fourth in July 2022, 227 days after the third dose; the fifth in December 2022, 147 days after the fourth dose; and the sixth in June 2023, 140 days after the fifth dose.

In October 2023, 161 days after the sixth dose, the patient received the seventh dose and subsequently experienced his first breakthrough infection 80 days after vaccination. The symptoms included fever, sore throat, cough, and fatigue. A SARS-CoV-2 antigen test conducted the day after symptom onset was positive. Lagevrio (800 mg) was administered twice daily for five days, resulting in symptom improvement within one week.

**Figure 1:**
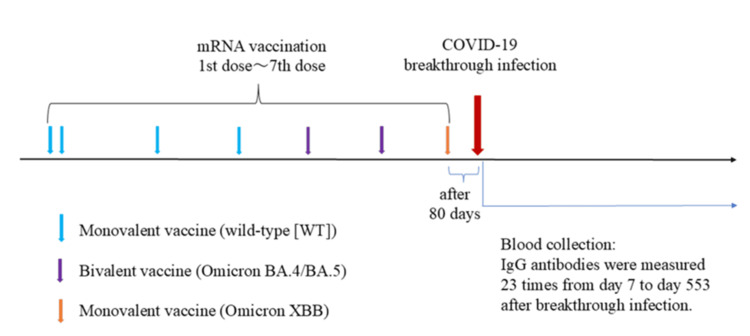
Timeline from the initial COVID-19 vaccination to breakthrough infection A breakthrough infection was observed 80 days following the administration of the seventh mRNA vaccination. Blood samples were obtained one week subsequent to the infection, and immunoglobulin (Ig)G antibody levels were assessed.

All seven doses were BNT162b2 vaccine doses. The first through fourth doses were the same monovalent vaccine (wild-type (WT)), the fifth and sixth doses were the same bivalent vaccine (containing Omicron BA.4 and BA.5), and the seventh dose was a monovalent vaccine (Omicron XBB). During the observation period preceding the breakthrough infection, three anti-nucleocapsid antibody tests were negative, confirming this as the first breakthrough infection (Figure 1).

Antibody levels were quantified using the Architect SARS-CoV-2 IgG II Quant assay (Abbott, USA), which quantitatively evaluates serum anti-spike protein IgG antibody concentrations. This assay exhibited exceptional performance, with a sensitivity of 98.3% (95% CI: 90.6-100.0%) and specificity of 99.5% (95% CI: 97.1-100.0%) [9]. Blood samples were collected periodically in accordance with the study design, and antibody levels were measured at a commercial laboratory (BML Inc., Japan). The cutoff level for this assay was 50 AU/mL. IgG antibody levels were measured before the initial administration of BNT162b2 and systematically assessed from the first to the sixth dose. No antibody tests were conducted after the seventh dose. In the event of breakthrough infection, antibody measurements were performed weekly for eight weeks and subsequently every two to six weeks, culminating in a total of 23 tests (Table 1).

**Table 1:** Antibody levels following a breakthrough infection after the seventh vaccination Antibody concentrations in all blood samples were assessed six times at weekly intervals commencing seven days post-infection, followed at intervals ranging from two to six weeks.

Days after breakthrough infection	Antibody titer (AU/ml)
7	5555
14	11000
21	34000
28	49000
35	55000
42	76000
48	74000
62	61000
83	60000
132	58000
160	44000
188	46000
216	37000
248	39000
272	30000
296	26000
334	26000
375	23000
403	15000
431	25000
462	23000
508	21000
553	19000

**Figure 2:**
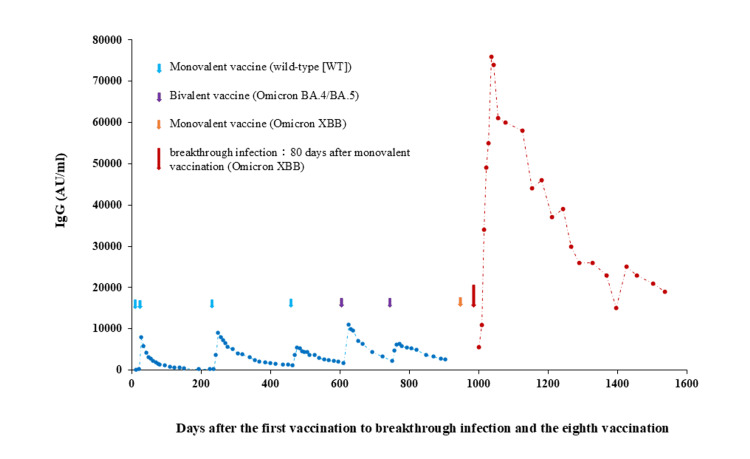
The study examined variations in IgG antibody levels in the patient Antibody levels were monitored following the first through sixth vaccinations and after a subsequent breakthrough infection. Notably, antibody levels post-seventh dose were not evaluated. The peak antibody levels were observed one to two weeks post-vaccination and six weeks post-breakthrough infection, after which an exponential decline was noted. Data pertaining to the first to sixth doses were sourced from reference [10]. IgG: Immunoglobulin G.

The sequential dynamics of anti-spike (RBD) IgG antibody levels from the initial vaccination through the sixth dose up to one year and eight months following breakthrough infection are depicted (Figure 2).

Antibody titers after doses 1-6 were reproduced from a previous study [10]. Following each vaccination, up to the sixth dose, antibody titers reached their peak at one to two weeks post-vaccination. However, antibody levels peaked at 76,000 (AU/ml) six weeks after breakthrough infection, approximately 14 times the baseline level (antibody titer one week post-infection). At one year and eight months post-infection, antibody titers remained at 19,000 (AU/ml), which is 3.8 times the baseline and one-fourth of the peak value, and approximately twice the peak values observed after each of the first to sixth vaccinations.

The alteration in IgG antibody titer following a breakthrough infection was estimated using a straightforward exponential equation: f(t) = Aexp(-*t*/τ) + C (where τ represents the time constant). This equation has been previously validated for modeling the antibody decay pattern post-booster vaccination [10]. In this instance, the coefficient of determination (R²), which serves as an indicator of the goodness of fit, was 0.93. The mean squared error (MSE) was calculated to be 24,187,982, the mean absolute error (MAE) was 4,238, and the root mean square error (RMSE) was 4,916.

y = 6.0 × 10^4^exp (-*t*/1.9 × 10^2^)+1.4 × 10^4^　 tmax/2 = 184 days　

**Figure 3:**
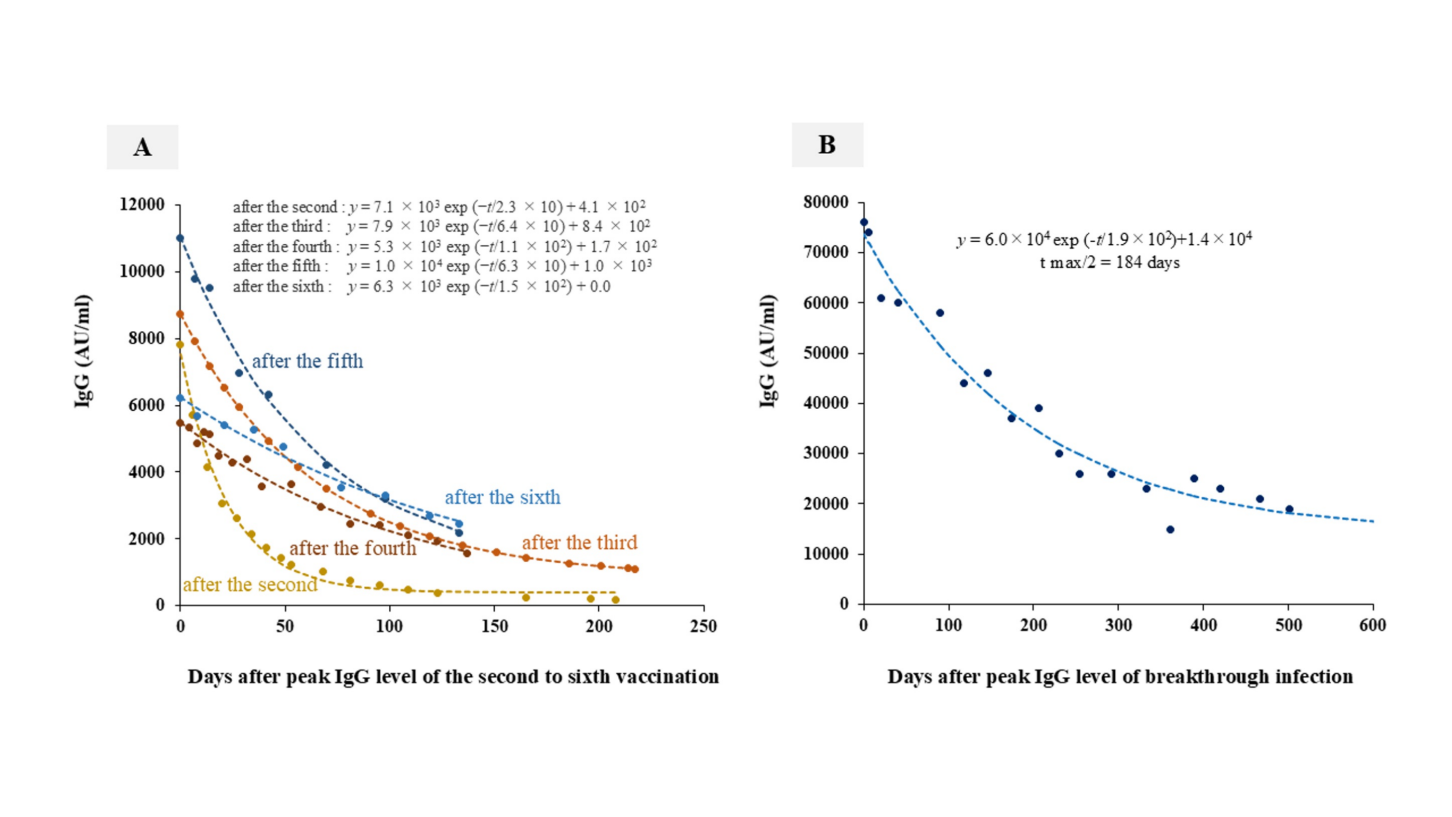
Exponential decay models were applied to data following the administration of booster doses of mRNA vaccines and subsequent to breakthrough infections The dynamics of immunoglobulin G (IgG) antibody levels following the administration of the second to sixth vaccine doses, as well as post-breakthrough infection, are illustrated through decay curves derived from approximate equations. The approximation equation is y = Aexp(−*t*/τ) + C, where τ denotes the time constant. Figure 3A presents the decay curves following the second to sixth vaccine doses, reprinted from reference [10], indicating a decrease in the decay rate with each successive dose. Figure 3B illustrates the decay curve following a breakthrough infection. The concentration of IgG antibodies exhibited an exponential decline from an initial peak of 76,000 AU/ml, with a half-life of 184 days, eventually stabilizing at 14,000 AU/ml.

Utilizing this exponential equation, the time required for the peak antibody titer to reduce by half (tmax/2) was determined to be 184 days. The antibody titer was measured at 14,000 AU/ml three years post-infection. The temporal progression of IgG antibody titers from their peak following breakthrough infection, along with the decay curve derived from the approximation equation, is depicted in Figure 3.

Similar to the second to sixth doses, the kinetics of antibody titer breakthrough infection also exhibited exponential decline.

## Discussion

The reduction in IgG antibodies following mRNA vaccination can be elucidated through a biphasic model characterized by an initial rapid decline followed by a more gradual decrease. The initial phase is predominantly attributed to a reduction in antibody production by short-lived plasma cells. Subsequently, the rate of antibody titer decline decelerates; at this juncture, antibody production by long-lived plasma cells and memory B cells becomes predominant [10].

The timing of antibody level assessment post-vaccination is of paramount importance in investigations of humoral immunity, as indicated by IgG antibody levels. The initial post-vaccination antibody titer is contingent upon the interval between vaccination and testing, typically reaching its peak one to two weeks after booster vaccination [10]. A comprehensive model for the attenuation of IgG titers after mRNA vaccination has not yet been developed. In the current study, the peak timing of IgG antibody levels following the second to sixth doses varied based on the vaccine type and number of doses administered. Specifically, the first doses of repeated administrations of the same vaccine, the second dose (first dose of wild-type (WT) vaccine), and the fifth dose (first dose of vaccines containing Omicron BA.4 and BA.5), peaked one week after administration. Conversely, for repeated doses of each vaccine, the third, fourth, and sixth doses peaked two weeks after administration [10]. As these variations in peak timing and their underlying immunological mechanisms have not been documented, they remain unknown. Although the attenuation following each vaccination adheres to an exponential decay pattern, longitudinal analysis of such patterns through frequent measurements is exceedingly rare [11].

In this study, we demonstrated that even after breakthrough infection following the seventh dose, the attenuation kinetics mirrored those observed after previous boosters. Several mathematical models concerning the decay of IgG antibodies have been proposed [12]; however, they are predicated on IgG antibody levels measured three to four times after vaccination. Consequently, establishing a mathematical model that accurately describes the long-term dynamics of IgG antibodies is challenging. As most individuals experience breakthrough infections at varying intervals, studying antibody dynamics longitudinally is difficult. This case report is the first to unequivocally demonstrate, over an extended period of four and a half years, that antibody titer dynamics following both frequent booster vaccinations and breakthrough infections exhibit exponential decay.

The administration of mRNA vaccines induces rapid and synchronized antigenic stimulation at the injection site, leading to a rapid peak in antibody titers. Conversely, during infection, continuous viral replication and sustained antigen presence result in a more gradual antibody response, with the peak potentially delayed owing to prolonged antigen supply [13]. In the current case, the peak antibody titer post-vaccination was achieved within one to two weeks, whereas it required approximately six weeks to reach peak levels following breakthrough infection.

A mathematical model analysis from a human challenge study involving COVID-19 (12 healthy young volunteers) demonstrated that viral RNA levels rapidly escalated to a peak (median of 6.5 days post-infection) before declining to an undetectable level (median of 16.5 days post-infection). This post-peak decline suggests that the emergence of adaptive immune mechanisms between days 7 and 11 post-infection is critical and contributes significantly to viral elimination and clearance [14].

In immunocompromised individuals with a history of hematologic malignancy or organ transplantation, the development of humoral immune responses, particularly neutralizing antibodies, is compromised, resulting in delayed viral RNA clearance (median, 72 days) and a strong association with persistent long-term infection [15]. In the present case involving an older individual, it is posited that immune responses are attenuated, potentially delaying post-infection viral clearance, which aligns with the observation of a significantly delayed IgG antibody peak compared to that post-vaccination. Thus, an inadequate humoral immune response is directly linked to insufficient viral clearance, and an individual's immunological background significantly influences studies on post-infection antibody dynamics.

A breakthrough infection was observed 80 days after the administration of the seventh dose of the monovalent vaccine (Omicron XBB). At the time of this breakthrough infection in January 2024, 77% of the COVID-19 variants circulating in Japan belonged to the JN.1 lineage, whereas other Omicron lineages, such as the XBB lineage, were rapidly declining [16]. Consequently, it is plausible that the efficacy of the seventh vaccination in this case was circumvented by immunity.

Post-mRNA vaccination anti-S protein IgG antibodies were highly correlated with neutralizing antibodies in the early stages of the pandemic. However, although IgG antibody levels remain preserved against Omicron variants, their neutralizing activity is significantly diminished [17]. It has been demonstrated that the measurement of neutralizing antibodies against Omicron variants is more critical than the evaluation of IgG antibody titers in determining vaccine efficacy.

Conversely, distinctions have been identified in the quality of immune responses and underlying immune memory between mRNA vaccines and breakthrough infections. Following booster vaccination, the antibody response predominantly arises from the reactivation of pre-existing memory B cells, with limited activation of new B cells specific to the Omicron variant [18]. A breakthrough infection after a booster dose has been shown to significantly increase the frequency of B cells encoding SARS-CoV-2 neutralizing antibodies. Moreover, during multiple antigen exposures, broadly reactive memory B cells develop early, offering cross-reactivity and cross-protection against novel variants that have not been previously encountered by the individual [18].

A systematic review of the protection from previous SARS-CoV-2 infection and the magnitude and duration of hybrid immunity (vaccine + infection) against infection and severe disease caused by Omicron variants indicated that all estimated protection against reinfection declined within a few months; however, high levels of protection against hospitalization and severe disease remained [19].

Currently, large-scale randomized controlled trials or systematic reviews to determine the optimal interval between additional doses following breakthrough infection are lacking, leaving the timing of such doses uncertain. However, it is anticipated that an interval of at least four months between vaccine doses may enhance cross-neutralization, which involves a broad antibody response against multiple variants. Consequently, it is deemed appropriate to allow sufficient intervals between doses after a breakthrough infection, with regular annual vaccinations considered reasonable [20], and individualized approaches are recommended.

However, long-term data on next-generation mRNA technologies for emerging variants are insufficient. The development and antigen design of new variant-adapted vaccines, as well as pancorona vaccines aimed at eliciting broadly protective immune responses against multiple coronavirus strains and lineages, continue to be significant areas of future research [4]. Additionally, large-scale cohort studies are necessary to ascertain the optimal dosing intervals and clinical effectiveness of hybrid immunity and heterologous vaccination.

This mathematical model of the kinetics of humoral immunity decay may prove beneficial for informing future mRNA vaccination strategies, particularly in the context of breakthrough infections following multiple booster vaccinations. The limitations of this case study encompass its nature as a single case report, the absence of pre-infection antibody testing, the lack of data to assess neutralizing activity, and the absence of confirmation regarding the mutated strain of the infecting virus.

## Conclusions

A 71-year-old patient experienced a breakthrough infection following the administration of the seventh dose (fifth booster dose) of the BNT162b2 vaccine. A total of 23 serological assessments were conducted to quantify anti-spike protein IgG antibody titers from one week to one year and eight months post-infection. Six weeks post-infection, the antibody titer reached a peak of 76,000 AU/ml, representing a 14-fold increase from the baseline value. Notably, even at one year and eight months post-infection, the antibody titer remained at 19,000 AU/ml, equating to 3.8 times the baseline value and one-quarter of the peak value. The kinetics of antibody titer decline after breakthrough infection were fitted to an exponential equation: y = 6.0 × 10^4 ^exp(-*t*/1.9 × 10^2^)+1.4 × 104, with a half-life of peak antibody levels of 184 days. To our knowledge, this case study is the first to employ a mathematical analysis of frequent antibody tests post-breakthrough infection, with no similar reports available in the literature. These findings indicate that humoral immune responses are sustained at a high level over an extended period. This case study offers significant insights into the dynamics of the humoral immune response in breakthrough infections following multiple booster vaccinations.
